# Persistent Low-Level Replication of SIVΔnef Drives Maturation of Antibody and CD8 T Cell Responses to Induce Protective Immunity against Vaginal SIV Infection

**DOI:** 10.1371/journal.ppat.1006104

**Published:** 2016-12-13

**Authors:** Sama Adnan, R. Keith Reeves, Jacqueline Gillis, Fay E. Wong, Yi Yu, Jeremy V. Camp, Qingsheng Li, Michelle Connole, Yuan Li, Michael Piatak, Jeffrey D. Lifson, Wenjun Li, Brandon F. Keele, Pamela A. Kozlowski, Ronald C. Desrosiers, Ashley T. Haase, R. Paul Johnson

**Affiliations:** 1 Yerkes National Primate Research Center, Emory University, Atlanta GA, United States of America; 2 New England Primate Research Center, Harvard Medical School, Southborough Campus, Pine Hill Drive, Southborough, MA, United States of America; 3 Center for Virology and Vaccine Research, Beth Israel Deaconess Medical Center, Boston, MA, United States of America; 4 Department of Microbiology, Immunology and Parasitology, Louisiana State University Health Sciences Center, New Orleans, Louisiana, United States of America; 5 Nebraska Center for Virology and School of Biological Sciences, University of Nebraska-Lincoln, Lincoln, Nebraska, United States of America; 6 AIDS and Cancer Virus Program, Leidos Biomedical Research, Inc., Frederick National Laboratory for Cancer Research, Frederick, Maryland, United States of America; 7 Department of Medicine, University of Massachusetts Medical School, Worcester, MA, United States of America; 8 Department of Pathology, University of Miami Miller School of Medicine, Miami, Florida, United States of America; 9 Department of Microbiology, Medical School, University of Minnesota, MMC 196, 420 Delaware Street S.E., Minneapolis, Minnesota, United States of America; 10 Ragon Institute of MGH, MIT and Harvard, Cambridge, MA, United States of America; National Institutes of Health, UNITED STATES

## Abstract

Defining the correlates of immune protection conferred by SIVΔnef, the most effective vaccine against SIV challenge, could enable the design of a protective vaccine against HIV infection. Here we provide a comprehensive assessment of immune responses that protect against SIV infection through detailed analyses of cellular and humoral immune responses in the blood and tissues of rhesus macaques vaccinated with SIVΔnef and then vaginally challenged with wild-type SIV. Despite the presence of robust cellular immune responses, animals at 5 weeks after vaccination displayed only transient viral suppression of challenge virus, whereas all macaques challenged at weeks 20 and 40 post-SIVΔnef vaccination were protected, as defined by either apparent sterile protection or significant suppression of viremia in infected animals. Multiple parameters of CD8 T cell function temporally correlated with maturation of protection, including polyfunctionality, phenotypic differentiation, and redistribution to gut and lymphoid tissues. Importantly, we also demonstrate the induction of a tissue-resident memory population of SIV-specific CD8 T cells in the vaginal mucosa, which was dependent on ongoing low-level antigenic stimulation. Moreover, we show that vaginal and serum antibody titers inversely correlated with post-challenge peak viral load, and we correlate the accumulation and affinity maturation of the antibody response to the duration of the vaccination period as well as to the SIVΔnef antigenic load. In conclusion, maturation of SIVΔnef-induced CD8 T cell and antibody responses, both propelled by viral persistence in the gut mucosa and secondary lymphoid tissues, results in protective immune responses that are able to interrupt viral transmission at mucosal portals of entry as well as potential sites of viral dissemination.

## Author Summary

Annually, more than two million people worldwide are infected with HIV, the virus that causes AIDS. Rhesus macaques can be infected with SIV, a close relative and ancestor of HIV, resulting in simian AIDS, recapitulating key aspects of human HIV infection. SIVΔnef, a live attenuated form of SIV, protects rhesus macaques from subsequent challenge with pathogenic SIV and is widely viewed as the most effective SIV vaccine. Here we demonstrate that SIVΔnef persistence during the vaccination period drives both cell-mediated and humoral immune response maturation. During the vaccination period, cell-mediated immune responses elicited by SIVΔnef target more conserved regions of the virus rendering immune escape more difficult. Furthermore, the localization of the cell-mediated immune responses is shifted over time from peripheral blood to sites of viral production that are rich in uninfected SIV target cells, thereby positioning cell-mediated immune responses where they are most needed after wild-type SIV challenge. Similarly, SIVΔnef persistence during the vaccination period also leads to the accumulation and maturation of the humoral immune response. Our findings highlight the unique capacity of persistent vaccines to elicit durable and effective immune responses against wild-type SIV challenge.

## Introduction

Despite the considerable resources committed to developing an effective HIV vaccine over the past three decades, this objective remains elusive. Recent failures of HIV vaccine trials to demonstrate protection against infection [1, 2] and the only marginal apparent efficacy demonstrated in another, in which the observed limited protection was associated with unanticipated immune correlates [3], have refocused the field on comprehensive efforts to identify the fundamental determinants of a protective vaccine-induced immune response. Although few models of spontaneous lentiviral control exist, long-term nonprogressors and live attenuated SIV (LASIV) vaccinated animals have both proved to be highly illuminating models regarding the requisite immune correlates of viral control. The most effective lentiviral vaccine to date, SIVΔnef, has demonstrated durable protection against both systemic [4, 5] and mucosal challenge routes [6–8], and against heterologous challenge virus [7, 8]. However, safety issues identified first in SIVΔnef infection in infant macaques [9–11], and subsequently in some adults [10], preclude attenuated lentivirus vaccination as a viable vaccine strategy for HIV. Nonetheless, studies to identify correlates of protection in this premier model of successful vaccine-induced protection against lentiviral challenge could certainly shed light on critical attributes of a vaccine to protect against HIV.

A hallmark of live attenuated SIV vaccines, including SIVΔnef, is that protection against wild-type SIV pathogenic challenge typically increases with time, plateauing at 15 to 20 weeks post vaccination [5, 12, 13]. However, the immune mechanisms that underlie this protection, and its maturation, have been hotly debated. SIVΔnef vaccination generates strong antibody responses [14, 15] and depletion of CD8 T cells does not abrogate protection induced by SIVΔ3 (which contains deletions in *nef*, *vpr* and the U3 region) in Mamu-A*01- macaques [16], implicating antibody responses as a potential mechanism of protection. Furthermore, recent studies of anti-Env antibody responses have described SIV-specific antibody-dependent cell-mediated cytotoxicity (ADCC) [15, 17] and antibody responses to trimeric gp41 concentrated by the neonatal Fc receptor (FcRn) in the vaginal and cervical epithelium [17] as temporal and anatomic correlates of the maturation of protection in SIVΔnef-vaccinated animals.

Similarly, SIV-specific CD4 and CD8 T cell responses have also been implicated in SIVΔnef-induced protection [7, 8, 18–20]. Fukazawa and colleagues have correlated the magnitude of the T cell response in lymph nodes during live attenuated SIV vaccination, which in turn correlated with persistent residual replication of the vaccine virus in this site, with protection against wild-type challenge [19]. We have since shown that increased anentropic specificity, i.e. CD8 T cell responses targeting highly conserved epitopes, correlates with maturation of protection during SIVΔnef vaccination [20]. Most recently, we have demonstrated that low-level persistent SIVΔnef stimulation generates a unique signature of expression of transcription factors in SIV-specific CD8 T cells [21]. Finally, studies using a related model involving immunization with an attenuated SHIV vaccine have correlated protection against wild-type challenge with CD8 T cell responses in the female reproductive tract [22].

In this study we sought to delineate the temporal immune correlates of protection associated with maturation of immunity induced by SIVΔnef vaccination by employing a comprehensive approach involving both longitudinal studies and cross-sectional, intensive tissue sampling studies. We conducted two large vaccine trials using identical viral stocks and the same vaginal challenge route to determine the immune responses that correlate with protection against mucosal wild-type SIV challenge. The first study was a longitudinal vaccine trial designed to ascertain the temporal kinetics of SIVΔnef-induced protection against wild-type SIV vaginal challenge, and the second was a cross-sectional study to assay for immune responses in mucosal and lymphoid tissues during the SIVΔnef vaccination period and after SIV challenge. To resolve the contributions to protection of varying immune components, we have quantitated SIVmac239Δnef and wild-type SIVmac251 viral loads using a discriminating set of primers and simultaneously assayed for serum and vaginal antibody responses, determined the number of infecting viral variants after challenge using single genome analysis, and analyzed the functionality, phenotype, specificity and tissue localization of CD8 T cell responses. In these complementary vaccine studies, we demonstrate that the extent of SIVΔnef replication in gut and lymphoid tissues drives CD8 T cell polyfunctionality, localization of CD8 T cell responses to the tissues, antibody accumulation and affinity maturation, all of which correlate with protection against wild-type SIVmac251 vaginal challenge.

## Results

### Protective immunity against vaginal challenge induced by SIVΔnef vaccination is time dependent

**Fig 1:**
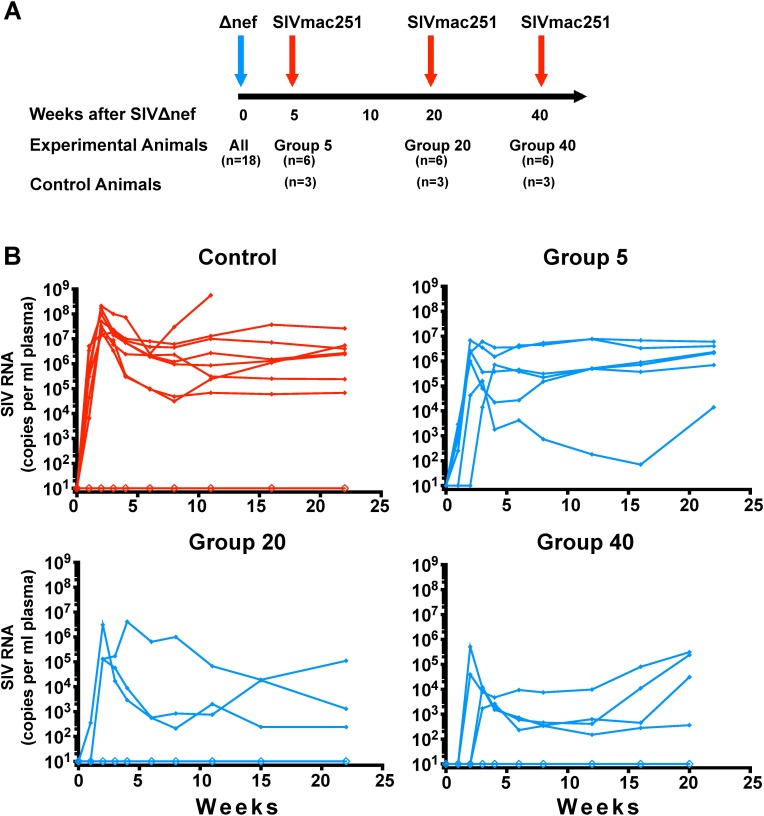
Timeline and outcome of the longitudinal vaccine study. (A) A schematic diagram demonstrating 18 animals were vaccinated with SIVΔnef and intra-vaginally challenged at week 5, week 20 or week 40 with SIVmac251. Three control animals were inoculated with SIVmac251 with each vaccination group. (B) Plasma viremia of control and SIVΔnef-vaccinated animals after vaginal challenge with SIVmac251.

To study the temporal immune correlates of SIVΔnef-induced protection against wild-type, SIVmac251 challenge, we intravenously vaccinated 18 female macaques with SIVmac239Δnef. MHC class I alleles associated with the control of SIV infection such as Mamu A*01 [23] and Mamu B*17 [24] were evenly distributed among experimental groups (S1 Table). Given that previous reports have documented increased protection during the first 20 weeks of SIVΔnef vaccination following intravenous challenge [5, 12, 13], the animals were challenged intravaginally with SIVmac251 at 5, 20 and 40 weeks post-vaccination to establish the kinetics of protection for this route of infection. As a control group, a total of nine unvaccinated animals were also intravaginally challenged with SIVmac251; three control animals were inoculated simultaneously with each of the three experimental groups (Fig 1A).

To discern between the viral loads of the SIVmac239Δnef vaccine and the SIVmac251 challenge virus, discriminating primers were used for the real-time quantitative PCR assay. Although all animals challenged 5 weeks after vaccination (Group 5 animals) were productively infected with wild-type SIV after intravaginal challenge with SIVmac251, nearly half of the animals in week 20 and week 40 groups manifested apparent sterile protection against this stringent high dose wild-type challenge, as assessed by plasma viremia (Fig 1B). Three of the animals vaccinated for 20 weeks and two of the animals vaccinated for 40 weeks with SIVΔnef displayed apparent sterile protection based on the lack of detectable wild-type plasma viremia after high-dose vaginal challenge. No such sterile protection was observed in the Group 5 animals, and all had measurable viral RNA as detected by RT-qPCR using SIVmac251-specific primers. Of the nine control animals challenged with SIVmac251, eight were productively infected.

**Fig 2:**
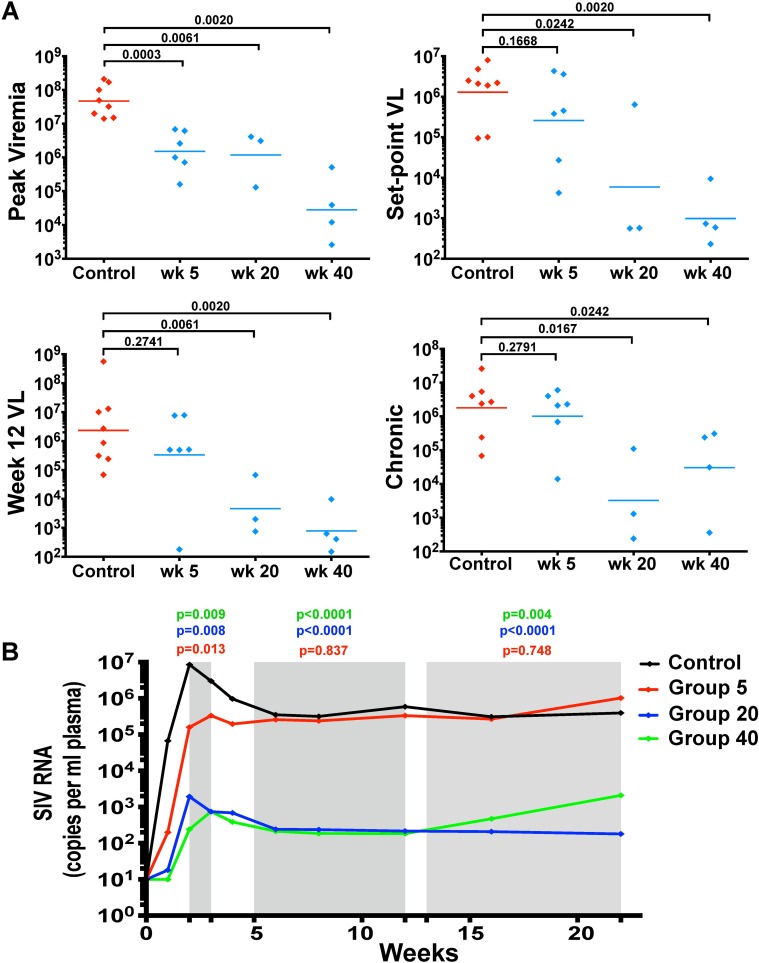
Lower viral loads in Group 20 and 40 SIVΔnef-vaccinated animals post-challenge. (A) Excluding animals that were sterilely protected in Groups 20 and 40, SIVΔnef-vaccinated animals infected at weeks 20 and 40 displayed sustained virological control during the first 20 weeks of challenge compared to control animals. In contrast, Group 5 animals only transiently suppressed SIV replication during peak viremia. Statistical tests conducted were all two-tailed Mann-Whitney tests. (B) Linear mixed model of plasma viral load for each group including all animals, infected and uninfected with wild-type challenge virus. The linear mixed model compared the viral loads of each vaccination group to the control group at three phases during the course of viral infection (shaded in grey): peak viremia, viral set-point (weeks 5 to 12), and chronic infection (weeks 13 to 22). Group 20 and 40 animals again demonstrate sustained virological suppression during the first 20 weeks post-challenge.

Despite significantly lower peak viremia for infected animals in all vaccinated groups compared to infected animals in the control group (p≤0.0061) (Fig 2A), set-point plasma viral load, measured at week 8 post-challenge, was similar for the eight infected, unvaccinated control animals and the six animals challenged at week 5 post-vaccination, at 1.3x10^6^ and 2.6x10^5^ RNA copies per ml of plasma, respectively (Fig 2A). In contrast, animals that were productively infected with SIVmac251 at week 20 or 40 post-vaccination had significantly lower set-point wild-type viral loads than control animals. At week 8 post-challenge, SIVmac251-infected animals in Groups 20 and 40 had an average set-point viremia of 5.9 X 10^3^ and 8.9 X 10^2^ RNA copies per ml of plasma, respectively (p = 0.024 and 0.002) (Fig 2A). Similarly, chronic viral loads in infected Group 20 and 40 animals, measured at weeks 16 and 20, were significantly lower compared to the Control Group (p≤0.0083 and p≤0.024, respectively) (Fig 2A).

A linear mixed model of post-challenge viral loads was used to determine the statistical significance of SIVΔnef vaccination for the three experimental groups compared to the control group, encompassing all animals whether infected or uninfected by the challenge virus. The linear mixed model allows for comparison between different vaccination groups based on the fixed effect of the duration of the vaccination period prior to pathogenic SIVmac251 challenge (5, 20 or 40 weeks), while taking effects such as animal genetic background into account. Statistical analysis was performed for three phases over the course of viral infection: peak viremia, viral set-point (weeks 5 to 12) and chronic infection (weeks 13 to 22). All three vaccinated groups had significantly lower peak viremia than the unvaccinated controls (p≤ 0.013) (Fig 2B). However, the Group 5 animals demonstrated only a transient reduction in viremia compared to unvaccinated animals and had comparable levels of viremia during the viral set-point and chronic phases of infection (Fig 2B). In contrast, Group 20 and Group 40 animals, including both partially and sterilely protected animals, had a significantly lower plasma viremia than the control group during acute infection (p<0.008), viral set-point (p<0.0001) and chronic infection (p≤0.004). We defined protection from SIVmac251 challenge in a given group as a greater than two-log viral load decrease in the first 20 weeks post-challenge compared to the control group. The protection of Group 20 and 40 animals but not Group 5 vaccinees demonstrates that SIVΔnef-induced protection is temporally determined and that a longer duration of vaccination correlates with protection from wild-type pathogenic mucosal challenge.

### SIVΔnef-vaccinated animals have fewer established viral variants

Having demonstrated disparate impact on plasma viremia among the different vaccination groups, we next asked whether the different levels of protection were also manifest as sieving effects on viral variants established in the disseminated systemic infection. Taking advantage of the fact that our challenge virus, SIVmac251, is a quasispecies and not a clone, we used single genome amplification [25] to estimate the number of transmitted viral variants that established the initial disseminated infection manifested as plasma viremia. Plasma viral RNA was reverse transcribed and the *env* gene in its entirety was amplified and sequenced for every animal. The sequences were then plotted as phylogenetic trees to determine the minimum number of viral variants that were transmitted and established in each animal.

**Fig 3:**
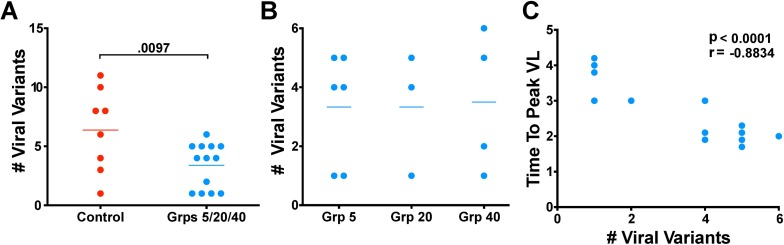
SIVΔnef vaccination reduces transmitted viral variants. (A) Vaccinated animals had significantly fewer viral variants than unvaccinated controls (p = 0.0097; two-tailed unpaired t-test) after inoculation with SIVmac251. (B) The number of transmitted viral variants did not differ between the unprotected Group 5 animals and the protected Group 20 and 40 animals. (C) In vaccinated animals, the time-to-peak viremia inversely correlated with the number of transmitted viral variants (p<0.0001, r = -0.8834; two-tailed Pearson correlation).

SIVΔnef vaccination had a significant effect on the number of SIVmac251 viral variants established after challenge. Whereas unvaccinated control animals had an average of six viral variants established after challenge, vaccinated animals with detectable wild-type SIV plasma viremia had an average of three established variants, a significant narrowing of the genetic bottleneck between unvaccinated and vaccinated animals (p = 0.0097) (Fig 3A). However, although SIVΔnef vaccination reduced the number of established variants compared to unvaccinated controls, the number of established variants was not inversely correlated with time from vaccination, and thus the reduction in established viral variants was not correlated with the temporal maturation of protection. Rather, the average number of variants establishing productive viral replication in infected animals was strikingly similar among the three vaccine groups, an average of 3.3 viral variants for Group 5 animals, 3.3 viral variants for Group 20 animals and 3.5 viral variants for Group 40 animals (Fig 3B).

The number of founder variants in the vaccinated animals across all groups did inversely correlate with the time to peak viremia (p<0.0001) (Fig 3C). This result, and the significant reduction in peak viremia even at 5 weeks, is consistent with protective mechanisms in vaccinated animals with some immediate impact on constraining systemic infection (and thus delaying peak viremia) that, nonetheless, require maturation for sustained effects on established systemic infection seen in set-point reductions at 20 and 40 weeks.

### SIV-specific antibody responses increase during the vaccination period

**Fig 4:**
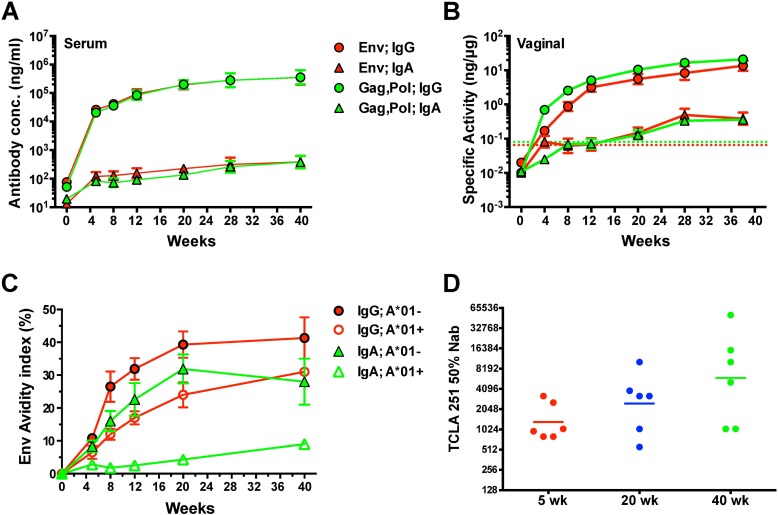
Prechallenge antibody responses in SIVΔnef-vaccinated animals. Concentrations of IgG and IgA binding antibodies to gp130 Env and Gag-Pol antigens in (A) serum and (B) vaginal secretions of all vaccinated animals were measured using ELISA. For vaginal secretions, the specific activity was calculated by dividing the IgG or IgA antibody concentration by the total IgG or IgA concentration in each sample. Shown are geometric means and SEM. The dashed line denotes the cut-off for significance (average specific activity + 3SD for negative controls). (C) The average avidity of Env-specific IgG and IgA antibodies in serum was determined using a NaSCN displacement ELISA. Error bars represent SEM. Mamu-A*01+ animals were found to have anti-Env IgG and IgA antibodies with significantly lower avidity than that in Mamu-A*01- animals. (D) A tissue culture lab adapted (TCLA) SIVmac251 was used to measure 50% neutralizing titers in serum of Grp 5, Grp 20 and Grp 40 animals on weeks 5, 20 and 40, respectively, after SIVΔnef vaccination. No significant differences were observed between the groups.

SIVΔnef induced significant IgG antibody responses in vaccinated animals as early as week 5 after vaccination. Serum IgG binding antibodies against Env and Gag-Pol rose quickly in the first 5 weeks after vaccination and continued to rise, albeit gradually, into week 40 (Fig 4A). Similarly, vaginal IgG antibody responses to Env and Gag-Pol rose precipitously in the first 5 weeks after vaccination and continued a slow increase into week 40 (Fig 4B). In both plasma and vaginal secretions, the SIV-specific antibody response was dominated by IgG responses, which were 2–3 logs higher than the SIV-specific IgA responses. The avidity of Env-specific serum IgG and IgA responses (Fig 4C), as well as the concentrations of anti-Env serum IgG responses (S1A and S1B Fig), were lower in Mamu-A*01^*+*^ animals compared to animals that did not express Mamu-A*01, probably due to the overall lower viral load levels achieved in Mamu-A*01^*+*^ animals during the vaccination period (S1C Fig).

In addition to binding antibodies and avidity, we also analyzed the ability of serum antibodies to inhibit infection of CEM cells by a neutralization-sensitive, T cell line adapted (TCLA) stock of SIVmac251. The titers of TCLA neutralizing antibodies in the serum of SIVΔnef-vaccinated animals doubled between weeks 5 and 20 and quadrupled between weeks 5 and 40 post-vaccination (Fig 4D). Serum TCLA neutralizing antibody titers also directly correlated with the cumulative antigenic load, calculated as the area under curve (AUC) of the plasma viral load (S2 Fig).

**Fig 5:**
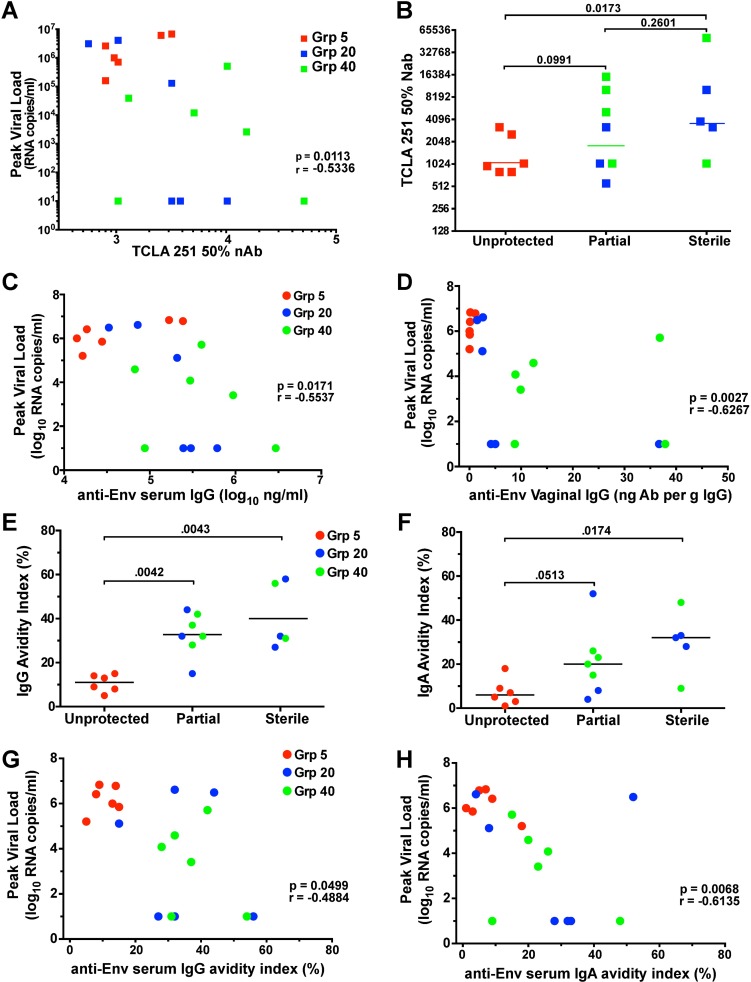
Humoral correlates of immune protection in SIVΔnef vaccinated animals. (A) TCLA SIVmac251 neutralizing antibody titers in serum on the day of challenge inversely correlated with peak post-challenge SIVmac251 viral loads. (B) TCLA neutralizing antibody titers in sterilely protected (though not partially protected) Grp 20/40 animals were significantly higher than those in unprotected Grp 5 animals. (C) Levels of anti-Env IgG binding antibodies in serum on the day of challenge and (D) anti-Env IgG specific activity in vaginal secretions collected 2 weeks before challenge, were inversely associated with peak post-challenge viremia. (E, F) The avidity of Env-specific serum IgG and IgA on the day of challenge was also significantly greater in sterilely protected animals. (G, H) Avidity of anti-Env serum antibodies in SIVΔnef vaccinated animals inversely correlated with peak post-challenge viremia. Two-tailed Spearman tests were used for all correlations and two-tailed Mann-Whitney tests were used for all comparisons between groups. Exclusion of the sterilely protected animals from the analysis presented in panel 5D also demonstrated a significant correlation between antibody titer and viral load (p = 0.049), while non-significant trends were noted for panels 5A, C, G and H when sterilely protected animals were excluded (p values = 0.076, 0.35, 0.36 and 0.074, respectively).

The TCLA neutralizing antibody titers in vaccinated macaques at time of challenge inversely correlated with peak SIVmac251 viremia after vaginal challenge (p = 0.011) (Fig 5A). While TCLA neutralizing antibody titers were significantly different between unprotected animals and those with apparent sterile protection, neutralizing antibody titers on the day of challenge were not significantly different between unprotected animals and partially protected animals, nor were they significantly different between partially protected animals and sterilely protected animals (Fig 5B). However, there was a trend toward higher TCLA neutralizing antibody titers between unprotected and partially protected animals and likewise between partially and sterilely protected animals.

Although levels of anti-Env IgA binding antibodies in serum and vaginal secretions did not correlate with peak viral load post-challenge (S3 Fig), post-challenge peak SIVmac251 viremia was inversely correlated to serum anti-Env IgG concentrations (p = 0.017) (Fig 5C) and demonstrated an even stronger inverse relationship to anti-Env IgG specific activity in vaginal secretions (p = 0.0027) (Fig 5D). The avidity of anti-Env serum antibodies on the day of challenge was also significantly greater in sterilely protected animals (Fig 5E & 5F), and it inversely correlated with peak viremia (Fig 5G & 5H), indicating that higher quality antibody responses were generated in both partially and sterilely protected animals. The inverse correlation between peak viremia and multiple parameters of SIV-specific antibody titers in both serum and the vaginal mucosa strongly support a role for humoral responses in SIVΔnef-induced protection.

### SIV-specific T cell responses increase in functionality

**Fig 6:**
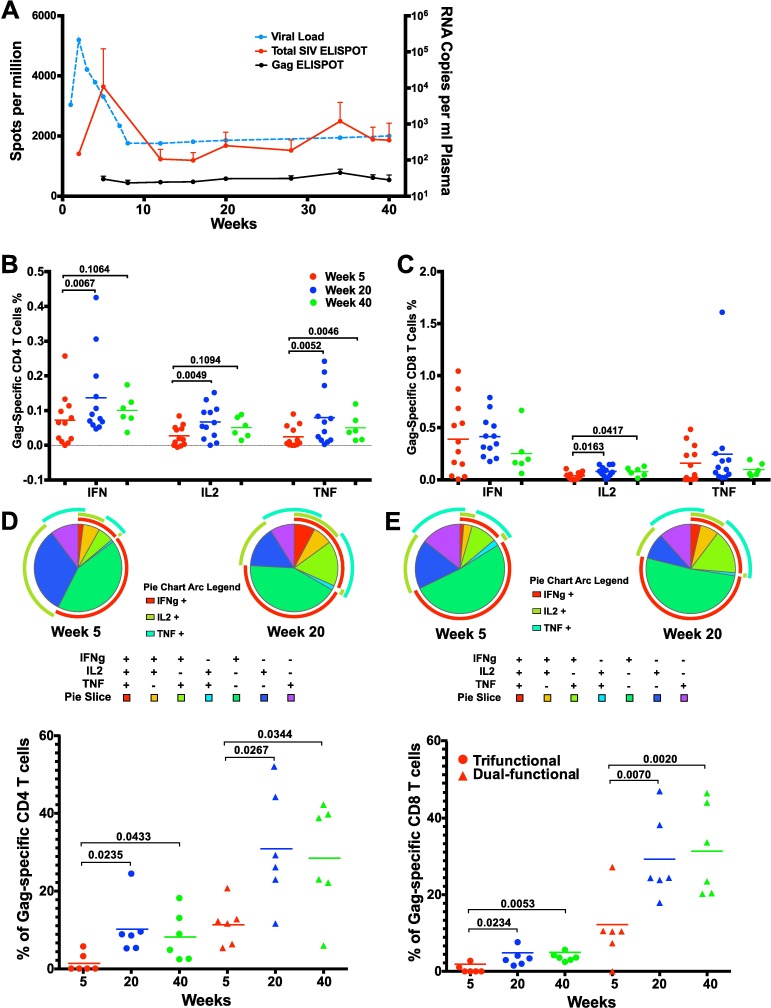
Functionality of SIV-specific T cell responses increased during SIVΔnef vaccination. (A) While the total SIV-specific T cell response as measured by IFN-γ ELISpot decreased during the first 20 weeks of SIVΔnef vaccination, the Gag-specific T cell response was maintained over time. Error bars reflect SEM. (B) Gag-specific CD4 T cells at week 20 expressed significantly higher levels of IFN-γ (p = 0.0067), IL-2 (p = 0.0049), and TNF-α (p = 0.0052) than Gag-specific CD4 T cells at week 5 (two-tailed paired t-test). (C) Gag-specific CD8 T cells at weeks 20 and 40 expressed significantly higher levels of IL-2 than at week 5 (two-tailed paired t-test). (D) Trifunctional Gag-specific CD4 T cell responses were significantly higher at week 20 (p = 0.0235) and week 40 (p = 0.0433) than at week 5; dual-functional CD4 T cell responses were also significantly higher at week 20 (p = 0.0267) and week 40 (p = 0.0344) (two-tailed Wilcoxon test). (E) Trifunctional Gag-specific CD8 T cell responses increased significantly between weeks 5 and 20 (p = 0.0234) and between weeks 5 and 40 (p = 0.0053); dual-functional CD8 T cell responses also increased significantly from week 5 to week 20 (p = 0.0070) and week 40 (p = 0.0020) (two-tailed Wilcoxon test).

Along with robust binding and TCLA neutralizing antibody responses, SIVΔnef also induced robust SIV-specific CD4 and CD8 T cell responses, as measured in blood. Following vaccination with SIVΔnef, plasma viremia peaked at week 2 post-inoculation and the total CD8 T cell response peaked at week 5 as measured by the IFN-γ ELISpot assay and declined following control of viremia (Fig 6A). However, although total SIV-specific CD8 T cell responses decreased between weeks 5 and 20, responses against the highly conserved Gag protein were maintained over time (Fig 6A), highlighting the importance of antigen specificity for CD8 T cell response persistence. Gag-specific CD8 T cell responses were also shown to be maintained between weeks 5 and weeks 20 and 40 as measured by IFN-γ and TNF-α expression using intracellular cytokine staining (ICS) assays, in addition to a significant increase in IL-2 production (p = 0.016) (Fig 6C). In contrast, IFN-γ-, TNF-α- and IL-2-producing Gag-specific CD4 T cell responses increased significantly between weeks 5 and 20 (p≤0.0067) (Fig 6B), indicating a significant upsurge in the functionality and magnitude of Gag-specific CD4 T cell responses at week 20. Similar trends of increased cytokine production by Gag-specific CD4 T cells between weeks 5 and 40 were also observed but were not statistically significant due to the small number of animals at week 40.

To ascertain the polyfunctionality of the T cell response at weeks 5 and 20, we stimulated peripheral blood mononuclear cells (PBMC) with overlapping Gag peptides and analyzed IFN-γ, TNF-α and IL-2 production. Using Boolean gating, we evaluated the functionality of Gag-specific CD4 T cells by determining the percentage that concurrently expressed all three cytokines or two of the three cytokines at weeks 5, 20 and 40 post-vaccination. The percentage of Gag-specific CD4 T cells expressing all three cytokines measured increased significantly between weeks 5 and 20 from 1.5 to 10 and between weeks 5 and 40 from 1.5 to 8.3 and, likewise, the percentage of Gag-specific CD4 T cells expressing 2 of the 3 cytokines assayed rose significantly from 12% at week 5 to 31% at week 20 and to 29% at week 40 (p≤0.043) (Fig 6D). Increased functionality in SIV-specific responses was also observed in CD8 T cells where the tri-functional Gag-specific CD8 T cells, coordinately expressing TNF-α, IFN-γ and IL-2, increased from 0.6% to 3.4% of total responses between weeks 5 and 20 (p = 0.023) and remained high at 3.8% up to week 40 (p = 0.0053). Similarly, Gag-specific CD8 T cell responses expressing 2 of the 3 cytokines comprised 11% of the response at week 5 and rose to 29% and 31% at weeks 20 (p = 0.0072) and 40 (p = 0.0020), respectively (Fig 6E).

Thus, in contrast to the declining magnitude of the total SIV-specific CD8 T cell response as detected by ELISpot assays to the entire SIV proteome, the magnitude of Gag-specific CD4 and CD8 T cell responses was maintained throughout the vaccination period. In addition, both Gag-specific CD4 and CD8 T cell responses increased in functionality over the forty-week SIVΔnef vaccination phase.

### SIVΔnef replication maintains CM9-specific CD8 T cell response in gut and lymphoid tissues

**Fig 7:**
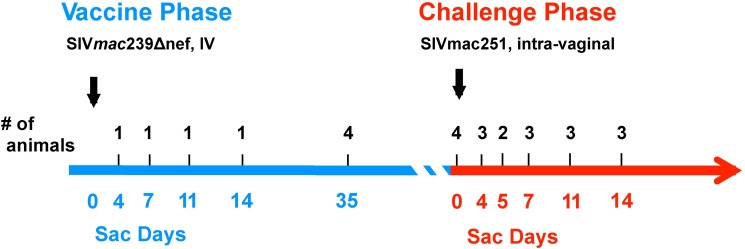
Schematic diagram of the cross-sectional serial sacrifice vaccine study. A total of 26 animals were vaccinated with SIVΔnef: four were sacrificed during the first two weeks of infection, four were sacrificed at week 5, and four sacrificed at week 20 (Challenge Day 0). The remaining 14 animals were challenged intravaginally with SIVmac251 and serially sacrificed at the indicated time points during the first two weeks after challenge.

Given that the magnitude of Gag-specific CD8 T cell responses as measured by cytokine production was maintained during the first 40 weeks of SIVΔnef vaccination, we sought to locate SIV-specific CD8 T cell responses within the female reproductive tract, the route of challenge; the gut mucosa, the anatomical site most enriched for activated CD4 memory T cells that are the most permissive target for SIV; and the secondary lymphoid tissues, where antigen-processing and presentation are most likely to occur after infection. In order to quantitate the localization, the magnitude and the specificity of SIV-specific CD8 T cells in the tissues during the SIVΔnef vaccination phase and the subsequent post-challenge phase, we conducted a follow-up cross-sectional serial sacrifice study. In the follow-up study, a total of 26 animals were vaccinated with SIVΔnef, of which 1 to 4 animals were sacrificed at days 4, 7, 11, 14, 35 and 140 post-vaccination. The remaining animals were challenged with SIVmac251 intravaginally and 2 to 4 macaques were sacrificed at days 4, 5, 7, 11 and 14 post-challenge (Fig 7).

Having demonstrated that animals vaccinated for 5 weeks with SIVΔnef were not protected compared to animals vaccinated for 20 or 40 weeks in the longitudinal study, we comprehensively examined animals at weeks 5 and 20 post-vaccination to determine the tissue localization of SIV-specific CD8 T cell responses. Tissues from secondary lymphoid tissues, including lymph nodes and the spleen; the gut mucosa, including the jejunum, the ileum and the colon; and the female reproductive tract, including the cervix and the vagina, were processed and stained for CM9-MHC tetramer^+^ and SL8-MHC tetramer^+^ CD8 T cells. CM9 is a highly conserved epitope within the Gag protein, while Tat SL8 is a highly variable epitope that escapes CD8 T cell responses mounted against it frequently and rapidly [20, 26]. These two epitopes on opposite extremes of the immune evasion spectrum were chosen to determine if epitope entropy, a measure of an epitope’s mutational flexibility, plays a role in the maintenance of its CD8 T cell response in the tissues.

**Fig 8:**
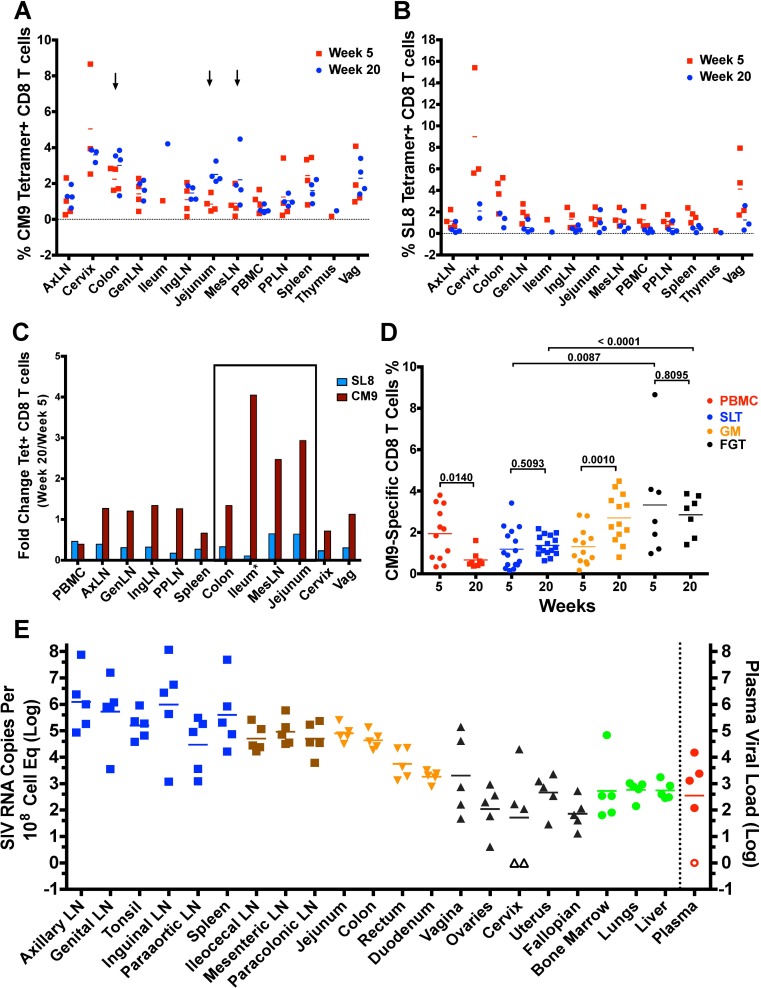
SIV-specific CD8 T cell response localizes to the gut and secondary lymphoid tissues during SIVΔnef vaccination. (A) CM9-specific CD8 T cells were maintained across tissues between weeks 5 and 20 post-SIVΔnef vaccination. Arrows highlight three tissues (colon, mesenteric lymph nodes and jejunum) where an increase in CM9-specific CD8 T cells is observed. (B) SL8-specific CD8 T cell responses wane in all tissues assayed. (C) The fold change of tetramer+ CD8 T cells between weeks 5 and weeks 20 shows that CM9-specific CD8 T cells were maintained or increased in every tissue except peripheral blood. *The fold change between weeks 5 and 20 for the ileum was based on one animal at each time point. (D) CM9-specific CD8 T cells were maintained in secondary lymphoid tissues (SLT) and the female genital tract (FGT), whereas they increased significantly in the gut mucosa (GM) (p = 0.0010) and decreased significantly in peripheral blood (PBMC) (p = 0.0143) (two-tailed unpaired t-test). (E) Comprehensive tissue and plasma viral quantitation reveals high levels of cell-associated viral RNA in secondary lymphoid tissues, the jejunum and the colon at 20 weeks post SIVΔnef-vaccination.

Overall, the frequency of CM9-specific CD8 T cell responses was maintained between weeks 5 and 20 in most tissues processed (Fig 8A). On the other hand, SL8-specific CD8 T cell response frequencies declined in every tissue processed (Fig 8B), confirming that epitope escape led to the waning of responding CD8 T cells in the tissues and that ongoing antigenic stimulation is necessary to maintain SIV-specific T cells in both lymphoid and non-lymphoid tissues. Looking at fold change of tetramer^+^ CD8 T cells at week 20 over week 5, SL8-specific CD8 T cell responses ranged between 0.1 and 0.65 in all tissues, signifying a 35–90% decrease between weeks 5 and 20, whereas CM9-specific CD8 T cell responses persisted at similar levels throughout most sites, with the notable exception of peripheral blood, where CM9-specific CD8 T cell responses experienced a similar drop to that of SL8-specific CD8 T cells (Fig 8C).

We also noted that the ileum, the jejunum, the colon and the mesenteric lymph nodes had increased frequencies of CM9-specific CD8 T cells from week 5 to week 20 post-vaccination. While CM9-specific CD8 T cell responses decreased significantly in peripheral blood (p = 0.014), their frequencies were maintained in secondary lymphoid tissues (p = 0.51) and the female genital tract (p = 0.81) and increased significantly in the gut mucosa (p = 0.001) (Fig 8D). Notably, the frequency of CM9-specific CD8 T cells was significantly higher in the female genital tract than it was in secondary lymphoid tissues at both week 5 (p = 0.0087) and week 20 (p<0.0001). To determine if the maintenance of CM9-specific CD8 T cell responses in secondary lymphoid tissues, the gut mucosa and the female genital tract was due to SIVΔnef replication in these tissues, we measured the cell-associated SIV RNA in these tissues at week 20. As has been previously shown, SIVΔnef replication persisted at relatively high levels in secondary lymphoid tissues (Fig 8E). Interestingly, the jejunum and the colon had levels of cell-associated viral RNA comparable to secondary lymphoid tissues. In contrast, peripheral tissues like the female genital tract, the lungs, liver and the rectum had very low cell-associated viral loads (Fig 8E).

**Fig 9:**
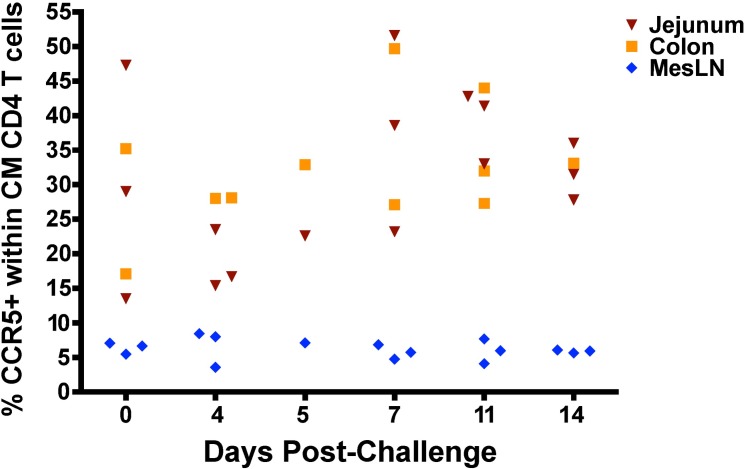
No depletion of CCR5+ central memory CD4 T cells in SIVΔnef-vaccinated animals after SIVmac251 challenge. SIVΔnef-vaccinated animals challenged with SIVmac251 at week 20 displayed no depletion of CCR5+ cells within the central memory CD4 T cell compartment in any gut tissues surveyed, including the jejunum, colon and mesenteric lymph nodes.

The presence of high frequencies of SIV-specific CD8 T cell responses in the gut, approaching 3% of total CD8 T cells for CM9-specific cells alone, highlighted the potential vaccination-induced protection of this important immune compartment and site of massive CD4 T cell depletion during wild-type SIV infection in unvaccinated animals. We phenotyped the CD4 T cell population in the jejunum, colon and mesenteric lymph nodes, all sites previously demonstrated to undergo massive depletion of CCR5+, central memory CD4 T cells during acute SIV infection [27]. In SIVΔnef-vaccinated animals subsequently challenged with wild-type SIVmac251 at 20 weeks post-vaccination, the frequency of CCR5+ CD28+ CD95+ CD4+ T cells did not decrease in the jejunum, the colon or the mesenteric lymph nodes between days 0 and 14 post-challenge (Fig 9). Nor was there a decrease of total memory CD4 T cells (CD95+ CD4+) in these tissues (S4A Fig) during the first 2 weeks post-challenge. Finally, the number of memory CD4 T cells as a percentage of CD3+ T cells in peripheral blood during the first two weeks was not different between uninfected and infected animals with the post-challenge SIVmac251 virus (S4B Fig), highlighting the prevention of CD4 T cell depletion in the gut after challenge even in animals that are partially, but not sterilely, protected.

### Tissue-resident SIV-specific CD8 T cell responses in the gut mucosa and female genital tract

**Fig 10:**
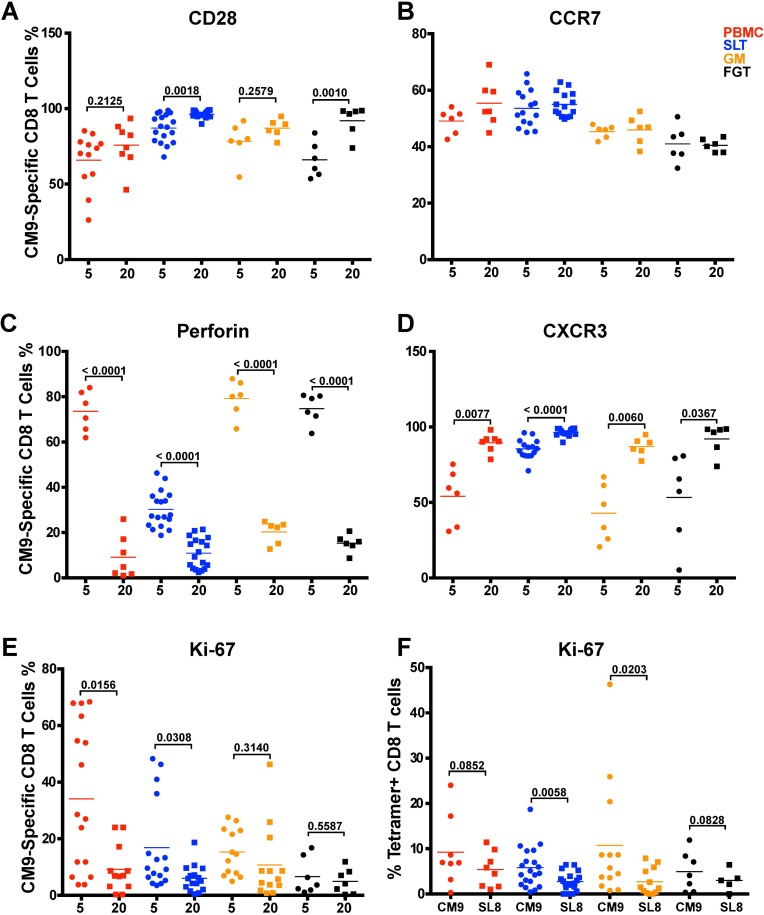
Maturation of CD8 T cell response to transitional memory phenotype during SIVΔnef vaccination. (A) Increased expression of CD28 in CM9-specific CD8 T cells in SLT (p = 0.0018) and FGT (p = 0.0010) between 5 and 20 weeks post-SIVΔnef vaccination. (B) No change in CCR expression between weeks 5 and 20 post-vaccination with SIVΔnef. (C) Significant decrease in perforin levels in CM9-specific CD8 T cells across all tissues (p<0.0001). (D) Increased expression of CXCR3 across all tissues between weeks 5 and 20 post-vaccination with SIVΔnef (p≤0.0367). (E) Significant decrease in Ki-67 levels in peripheral blood (p = 0.0156) and SLT (p = 0.0308) but no decrease in GM and FGT. (F) Ki-67 expression levels were higher in CM9-specific CD8 T cells than in SL8-specific CD8 T ells in SLT (p = 0.0058) and in GM (p = 0.0203) but not in peripheral blood or FGT. All statistical analyses were conducted using two-tailed unpaired t-tests.

Having demonstrated that SIV-specific CD8 T cell responses were maintained in the tissues, we phenotyped these responses using memory and functional markers over the 20-week vaccination period. Secondary lymphoid tissues and female genital tract CM9-specific CD8 T cells expressed higher levels of CD28 on the cell surface at week 20 than at week 5 (p = 0.0005, p = 0.001), denoting a shift to a more quiescent memory phenotype at week 20 (Fig 10A). Although CD28 expression levels in gut mucosa CM9-specific CD8 T cells were already high at week 20, there was no significant increase in the levels of CD28 expression in gut mucosa CM9-specific CD8 T cells between weeks 5 and 20, suggesting differences in the extent of antigenic stimulation in the different tissues (Fig 10A). In contrast, CCR7 expression levels in CM9-specific CD8 T cells remained relatively constant across all tissues and peripheral blood between weeks 5 and 20 (Fig 10B). Furthermore, in line with a shift from an effector memory cell to a more quiescent memory CD8 T cell response from week 5 to week 20, intracellular perforin levels in CM9-specific CD8 T cells decreased significantly between weeks 5 and 20 across all tissue compartments and peripheral blood (p<0.0001) (Fig 10C). Increased localization of CM9-specific CD8 T cells from the periphery to the tissues between weeks 5 and 20 (Fig 8D) was correlated with significantly increased expression of CXCR3 in CM9-specific CD8 T cells in all tissues and peripheral blood (p≤0.0124) (Fig 10D).

To determine if the sustained levels of CM9-specific CD8 T cells in the tissues were due to cellular proliferation, we assayed the intracellular expression of the proliferation marker Ki-67. While the expression of Ki-67 fell significantly in CM9-specific CD8 T cells in peripheral blood between weeks 5 and 20 (p = 0.0033), it declined less significantly in secondary lymphoid tissues (p = 0.0146) and did not decrease in the gut mucosa and the female genital tract (Fig 10E). Comparing Ki-67 expression levels at week 20 for the escaped SL8-specific CD8 T cell response and the CD8 T cell response specific to the highly conserved CM9, we observed similar percentages of cells expressing Ki-67 in peripheral blood. However, Ki-67 expression was significantly higher in CM9-specific CD8 T cells than in SL8-specific CD8 T cells in the secondary lymphoid tissue (p = 0.0058) and the gut mucosa (p = 0.02) (Fig 10F). The higher level of Ki-67 in tissue-resident CM9-specific CD8 T cells, but not CD8 T cells targeting SL8, likely reflects continued viral replication in the gut mucosa and the secondary lymphoid tissues that may explain both the maintenance of CM9-specific CD8 T cells.

**Fig 11:**
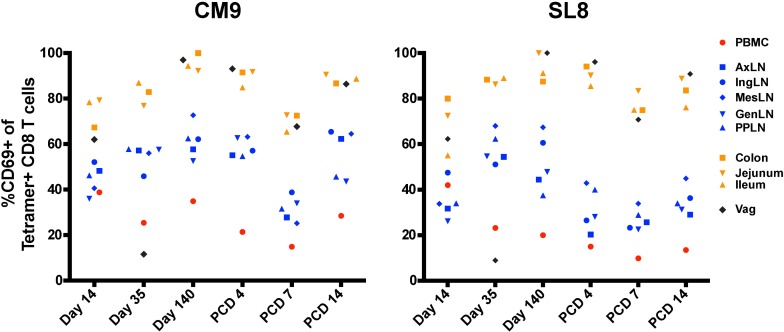
SIV-specific CD8 T cells express high levels of CD69 in gut and vaginal mucosae. Higher expression of CD69 in CM9-specific CD8 T cells and SL8-specific CD8 T cells in the vagina and the gut mucosa compared to secondary lymphoid tissues after SIVΔnef vaccination. (PCD: post-challenge day).

A number of recent reports have identified a distinct population of antigen-specific tissue resident CD8 T memory cells that do not recirculate and provide long-lived immunity against a variety of pathogens [28–30]. Although definitive phenotypic markers for tissue-resident memory have not yet been identified, expression of CD69 has been consistently observed in multiple studies [31, 32]. Upregulation of the activation marker CD69 has been demonstrated to block tissue egress of tissue-resident memory CD8 T cells by interfering with sphingosine-1-phosphate receptor (S1PR1) [33], and tissue-resident memory CD8 T cells express high levels of CD69 in the absence of recent activation [34–36]. To determine if SIV-specific CD8 T cells in nonlymphoid tissues displayed characteristics of stable populations of CD8 TRM, we phenotyped CM9- and SL8-specific CD8 T cells for CD69 expression. By week 20 after SIVΔnef vaccination, almost all CM9- and SL8-specific CD8 T cells in the jejunum, the colon, the ileum and the vagina upregulated CD69, in contrast to CD8 T cell responses in secondary lymphoid tissues and peripheral blood which were largely CD69- (Fig 11). Importantly, CD69 upregulation was not dependent on antigenic exposure, as the SL8 epitope is generally entirely escaped by week 5 [20, 26] nor was it a sign of CD8 T cell proliferation, as SIV-specific CD8 T cells in vaginal tissue demonstrated low Ki-67 expression at weeks 5 and 20 (Fig 10E, S5 Fig). Although SL8-specific CD8 T cells in gut and vaginal tissues also upregulated CD69 expression, the overall magnitude of the SL8 response dropped significantly in these tissues between weeks 5 and 20 (Fig 8C).

## Discussion

The combined data from these two SIVΔnef vaccine studies offer a number of novel findings that demonstrate the complex interplay of adaptive immune responses that ultimately result in the protection of SIVΔnef-vaccinated animals from wild-type vaginal challenge. The longitudinal vaccine study illustrates the protective effect of SIVΔnef vaccination against a minimally heterologous, high dose vaginal challenge with SIVmac251. SIVΔnef vaccine-induced protection against vaginal challenge, whether sterile or partial, was temporally dependent, increasing during the first 20 weeks of vaccination and sustained to 40 weeks post-vaccination. This time course is in contrast to SIVΔnef-induced protection against homologous intravenous challenge with SIVmac239, in which apparent sterile protection was observed as early as 5 weeks after vaccination [37]. SIVΔnef vaccination created a genetic bottleneck by reducing the number of established wild-type viral variants, which inversely correlated with a longer time-to-peak viremia. Both neutralizing and binding antibody titers increased during the vaccination period, and titers at time of challenge inversely correlated with peak viral load, while Gag-specific CD4 and CD8 T cell functionality increased in peripheral blood. The cross-sectional vaccine study reveals the preservation of CM9-specific CD8 T cell responses in high viral-load tissues, primarily in secondary lymphoid tissues and the gut mucosa, even as SL8-specific CD8 T cell responses waned, highlighting the importance of ongoing antigenic stimulation to maintain tissue-resident memory cells. Moreover, the persistent expression of Ki-67 in CM9-specific CD8 T cells in the secondary lymphoid tissues and the gut mucosa correlated with the maintenance of responses in these tissues, reinforcing the link between persistent local replication of the vaccine virus, associated antigenic stimulation and persistence of immune responses. In vaginal tissues, high CD69 expression coupled with low Ki-67 expression by SIV-specific CD8 T cells in the context of low viral replication strongly suggests the maintenance of a stable tissue-resident memory CD8 T cell population. Taken together, these results document induction of a highly functional immune response by SIVΔnef, employing each arm of the adaptive immune response and localizing to key sites of potential CD4 T cell infection.

Given the high efficiency of SIV infection of naïve controls following vaginal challenge using the SIVmac251 stock and challenge conditions employed in this study, which is consistent with previous studies with this stock [38, 39], the apparent sterile protection observed in the Group 20 and 40 animals is likely to represent a vaccine-mediated effect reflecting the maturation of protective cellular and humoral immune responses. Other potentially confounding factors, such as the menstrual cycle of the animals, appear unlikely to affect our results. Although there is evidence that the timing of the menstrual cycle may affect the susceptibility of macaques to low-dose SIV or SHIV challenge [40, 41], this effect has not been demonstrated in the setting of high dose SIV vaginal challenge. Given these considerations, as well as the fact that our animals were likely to be cycling asynchronously, it appears improbable that the differences in protection among the different experimental groups could be attributed to variations in the menstrual cycle of individual animals.

The effects of SIVΔnef vaccination on post-challenge virological outcome may be categorized as acute virological suppression, sustained virological suppression, and apparent sterilizing immunity. Acute virological suppression was characterized by a lower peak viral load, fewer established viral variants and a prolonged time-to-peak viremia, but was not temporally dependent. The reduction of viral variants was not sufficient to induce protection from challenge, since all groups of vaccinated animals had similar levels of established viral variants. By significantly decreasing peak viremia, perhaps a result of the decreased established number of viral variants, SIVΔnef limited the initial impact of wild-type SIV infection even at early time points after vaccination.

At later time points, protection against wild-type SIV vaginal challenge, whether partial or sterile, correlated with immune maturation of humoral and cellular immune responses. First, neutralizing antibody titers against TCLA SIV at day of challenge inversely correlated with peak wild-type SIV viral load. Second, in Mamu A*01+ animals the magnitude of the CM9-specific CD8 T cell response was largely maintained across most tissues during the vaccination period, even as SL8-specific responses declined to the limit of detection in all tissues assayed. This shift in specificity of the CD8 T cell response corroborates our previous results demonstrating increased anentropic specificity, characterized by the accumulation of CD8 T cell responses to highly conserved epitopes between weeks 5 and 20 post-SIVΔnef vaccination [20]. Furthermore, the difference in the persistence of CM9- and SL8-specific CD8 T cell responses can be attributed in part to increased proliferation of CM9-specific CD8 T cells. These results reinforce the concept that ongoing viral replication of SIVΔnef maintains the antigenic stimulation of CD8 T cell responses directed at conserved epitopes.

In addition to the shift in specificity refocusing the immune response on more conserved epitopes during the vaccination period, the CD8 T cell response was redistributed among tissues by week 20. During this time, the magnitude of CM9-specific CD8 T cells significantly increased in the gut mucosa and was maintained in secondary lymphoid tissues and the female genital tract, while CM9-specific CD8 T cell responses in peripheral blood significantly declined. At week 20, Ki-67 expression was significantly higher in CM9-specific CD8 T cells than in SL8-specific CD8 T cells in the gut mucosa and in secondary lymphoid tissues, suggesting that the observed viral replication in these tissues stimulated CM9-specific CD8 T cell proliferation. Meanwhile, tissue-resident memory CD8 T cells, which expressed high levels of CD69 and low levels of Ki-67, populated the vaginal tissues at week 5 and 20 post-SIVΔnef vaccination. Residual viral replication in gut and lymphoid tissues is likely to account for the transitional memory phenotype of CM9-specific CD8 T cells compared to SL8-specific CD8 T cells.

Fukazawa and colleagues have previously reported that the magnitude of T cell responses in secondary lymphoid tissues in animals vaccinated with different LASIV strains predicts protection from wild-type SIV challenge [19]. Our current work extends these findings to the gut mucosa. Comprehensive SIVΔnef RNA quantitation in tissues demonstrated that the gut mucosa, particularly the jejunum and colon, were important sites of SIVΔnef replication at 20 weeks after vaccination, producing comparable levels of viral RNA to secondary lymphoid tissues. The same factors that contribute to increased SIV replication in lymph nodes, namely the presence of B cell follicles, which contain easily infectable follicular CD4 T helper cells [19] and represent an immuneprivileged site impeding entry of SIV-specific CD8 T cells [42], are also found in Peyer’s patches in the lamina propia of the gut mucosa [43]. Sites of viral replication such as B cell follicles that are relatively protected from immune surveillance may be responsible for the ongoing antigenic stimulation required for protection induced by SIVΔnef.

In addition to shifting the specificity and tissue localization of the CD8 T cell response, persistent replication of SIVΔnef is likely to promote increased production and affinity maturation of antiviral antibodies, resulting in higher antibody affinity and higher neutralizing antibody titers. Antibody responses also directly correlated with SIVΔnef viral load within a given vaccination group, indicating that antigenic load and viral persistence play a key role in driving the induction and the affinity maturation of the antibody response.

Several observations indicate antibodies play an important role in preventing or suppressing replication of wild-type challenge virus in SIVΔnef-vaccinated animals. SIVΔnef-vaccinated animals with sterile protection had higher avidity Env-specific antibodies in serum on the day of challenge, and in all vaccinated animals, antibody avidity was inversely associated with peak post-challenge viremia. Serum anti-Env IgG binding antibody levels and TCLA neutralizing titers also demonstrated a strong inverse correlation with peak plasma viremia, consistent with prior observations of SIV-specific ADCC activity in this cohort of SIVΔnef-vaccinated animals [15]. Moreover, anti-Env binding antibody titers in the vaginal mucosa showed an even stronger inverse correlation with peak viral load post-challenge, suggesting that SIV-specific antibody responses inhibit viral transmission and spread in the female reproductive tract. The importance of SIV-specific responses localized to the female reproductive tract in mediating protection against vaginal challenge induced by SIVΔnef is also reinforced by our observations of a temporal maturation of antibodies to trimeric gp41 that are concentrated by the neonatal Fc receptor (FcRn) in the vaginal mucosa and cervical epithelium prior to [17] and following challenge [44]. Our results, suggesting a role for antibody responses during the early stages of wild-type SIV vaginal challenge, corroborate our previous findings [15] that ADCC activity in SIVΔnef-vaccinated animals exhibited a trend towards a negative correlation with post-challenge peak viral load. While our previous study did show a significant relationship between ADCC and apparent sterile protection, we did not detect any neutralizing antibody responses against the difficult-to-neutralize SIVmac251 stock in SIVΔnef-vaccinated animals on the day of challenge. In the present study, however, neutralizing antibodies against TCLA SIVmac251 did significantly correlate with early viral control post-SIV vaginal challenge, in addition to being significantly higher in sterilely protected animals than unprotected animals.

Collectively the results of our longitudinal and cross-sectional analysis of the maturation of protection are consistent with mechanisms of immune control that operate at different stages of infection. In the early stages of infection following wild-type SIV vaginal challenge, the immediate effects of antibody responses and CD8 T cell responses are likely to account for the reduction of viral variants and the delay in time-to-peak viremia. However, sterilizing immunity or significant reduction in set point plasma viremia require an immunologically matured response that impacts systemic infection. At subsequent stages when virus has disseminated to the gut and secondary lymphoid tissues, control of systemic infection requires a functionally mature CD8 T cell response focused on conserved epitopes. This pattern of immune protection induced by SIVΔnef differs significantly from that induced by recombinant CMV vectors, which induce early control and subsequent clearance of SIV infection in about 50% of animals but do not result in reduction in set-point viremia in vaccinated animals with established infection [45, 46].

Studies on SIVΔnef, the most effective vaccine to date against lentiviral challenge, continue to provide important insights as to how to design a successful HIV vaccine. SIVΔnef persistence drives the response specificity to conserved epitopes, redistributes the CD8 T cell responses to CD4-rich gut and lymphoid tissues, replenishes the SIV-specific resident memory CD8 T cell population in vaginal tissues, and induces increased antibody production and affinity maturation. Future studies on SIVΔnef will be required to tease out the relative contributions of CD8 T cell responses and neutralizing antibodies to apparent sterilizing immunity and long-term viral control. Taken together, these findings highlight the importance of developing clinically applicable vaccine strategies to provide ongoing antigenic stimulation that induces the maturation and localization of both humoral and cellular responses able to prevent lentivirus infection.

## Materials and Methods

### Ethics statement

The animals included in this study were all female Indian-origin rhesus macaques (*Macaca mulatta*), housed in a biocontainment facility at the New England Primate Research Center (NEPRC). MHC class I genotypes were determined by sequence-specific PCR [47]. These experiments and procedures were approved by the Harvard Medical Area Institutional Animal Care and Use Committee (the Harvard Medical Area Standing Committee on Animals), protocol 04383. The Harvard Medical School animal management program is accredited by the Association for the Assessment and Accreditation of Laboratory Animal Care, International (AAALAC), and meets National Institutes of Health standards as set forth in the 8^th^ edition of the Guide for the Care and Use of Laboratory Animals [48]. The institution also accepts as mandatory the PHS Policy on Humane Care and Use of Laboratory Animals by Awardee Institutions and NIH Principles for the Utilization and Care of Vertebrate Animals Used in Testing, Research, and Training. There is on file with the Office of Laboratory Animal Welfare (OLAW) an approved Assurance of Compliance (A3431-01).

All animals were housed indoors in an SOP-driven, AAALAC-accredited facility. Husbandry and care met the guidance of the Animal Welfare Regulations, OLAW reporting and the standards set forth in The Guide for the Care and Use of Laboratory Animals. All research animals were enrolled in the NEPRC behavioral management program, including an IACUC-approved plan for Environmental Enrichment for research primates. This program included regular behavioral assessments, and provision of species appropriate manipulanda, and foraging opportunities. This protocol had an IACUC-approved exemption from social housing based on scientific justification. Primary enclosures consisted of stainless steel primate caging provided by a commercial vendor. Animal body weights and cage dimensions were regularly monitored. Overall dimensions of primary enclosures (floor area and height) met the specifications of The Guide for the Care and Use of Laboratory Animals, and the Animal Welfare Regulations (AWR's). Further, all primary enclosures were sanitized every 14 days at a minimum, in compliance with AWRs. Secondary enclosures (room level) met specifications of The Guide with respect to temperature, humidity, lighting and noise level. The animals were provided ad lib access to municipal source water, offered commercial monkey chow twice daily, and offered fresh produce a minimum of three times weekly. Light cycle was controlled at 12/12 hours daily. The animals were subject to twice daily documented observations by trained animal care and veterinary staff, and enrolled in the facility's environmental enrichment, and preventative health care programs. Euthanasia took place at defined experimental endpoints using protocols consistent with the American Veterinary Medical Association (AVMA) guidelines. Animals were first sedated with intramuscular ketamine hydrochloride at 20 mg/kg body followed by sodium pentobarbital (≥100 mg/kg) intravenously to achieve euthanasia.

### SIVΔnef vaccination and SIVmac251 vaginal challenge

Animals were intravenously vaccinated with SIVmac239Δnef using either 5 ng or 25 ng of SIVp27. Vaginal challenges were carried out using 1.0 ml of SIVmac251 (kindly provided by Chris Miller, University of California-Davis) inoculated twice in a single day, with a 4 hour interval between inoculations. This stock of SIVmac251 (SIVmac251-CM) contains approximately 2 × 10^9^ viral RNA copies/ml and approximately 5 × 10^4^ 50% tissue culture infectious dose 50% (TCID_50_)/ml [49]. This vaginal challenge regimen and stock has previously resulted in highly efficient infection of naïve controls [38, 39]. At the time of vaginal challenge, animals were singly housed and assumed to be cycling asynchronously. Due to the requirement of performing vaginal challenges at defined times after SIVΔnef vaccination, it was not possible to perform vaginal challenges in relation to the menstrual cycle of individual animals.

### Quantitative viral RNA analysis

For quantitation of plasma viral loads in SIVΔnef-infected animals, highly specific, real-time RT-PCR assays were performed as described previously [50]. The assay specific for SIVmac239Δnef was developed by designing a reverse primer that recognizes the new sequence generated by the deletion of the *nef* gene. Viral RNA loads for animals infected with SIVmac251 were determined through highly specific, real-time RT-PCR assays using a specific reverse primer, which binds to the *nef* sequence in SIVmac251 but not SIVmac239Δnef. The nominal threshold for these assays was 30 viral RNA copy equivalents/ml plasma. Tissue viral load analysis was conducted as previously described [45].

### Single genome amplification

Single genome amplification to determine the number of transmitted viruses was conducted as described previously [25, 51]. Briefly, RNA was isolated using viral RNA extraction kit (Qiagen) and cDNA synthesized using gene specific priming and super script III (Life Technology). The cDNA was diluted in 96-well plates such that fewer than 29 PCRs yielded an amplification product. First-round PCR was carried out in 1× High Fidelity platinum PCR buffer, 2 mM MgSO_4_, 0.2 mM of each deoxynucleoside triphosphate, 0.2 μM of primers in *vif* and *nef*, and 0.025 U/μl platinum Taq High Fidelity polymerase (Invitrogen, Carlsbad, CA) in a 20-μl reaction mixture. The PCR mixtures were set up in MicroAmp optical 96-well reaction plates (Applied Biosystems, Foster City, CA) and sealed with ABI MicroAmp adhesive film. The following PCR conditions were used: 94°C for 2 min followed by 35 cycles of 94°C for 15 s, 55°C for 30 s, and 68°C for 4 min, with a final extension of 68°C for 10 min. Second-round PCR was carried out using 1 μl of the first-round product and 0.2 μM of a primer set spanning the *env* gene with the same PCR mixture as the first round. The PCR conditions included: 94°C for 2 min followed by 45 cycles of 94°C for 15 s, 55°C for 30 s, and 68°C for 4 min, with a final extension at 68°C for 10 min. The amplicons were sized on precast 1% agarose E-gel 96 (Invitrogen Life Technologies, Carlsbad, CA). All products derived from cDNA dilutions yielding less than 30% PCR positivity were sequenced.

### DNA sequencing

Viral *env* genes were sequenced by using BigDye Terminator chemistry and the protocols recommended by the manufacturer (Applied Biosystems, Foster City, CA). The sequences were determined by using an ABI 3730xl genetic analyzer (Applied Biosystems, Foster City, CA) and edited by using the Sequencher program, version 4.7 (Gene Codes, Ann Arbor, MI). Both strands of DNA were sequenced. All chromatograms were carefully inspected for sites of ambiguous sequence (double peaks), and those that contained one or more positions of mixed bases were excluded from further analysis.

### Measurement of SIV-specific antibody responses

ELISA was used to measure antibodies in serum and in vaginal secretions collected with Weck-Cel sponges as previously described [52, 53]. All samples intended for IgA analysis were first depleted of IgG using Protein G sepharose (GE Healthcare) as described [53]. Briefly, microtiter plates were coated with either recombinant SIVmac239 gp130 envelope (Env) protein (ImmunoDiagnostics, Woburn, MA) or SIVmac239 viral lysate (Advanced Biotechnologies Inc, Columbia, MD). Because the lysate lacks detectable Env protein at the 1/400 coating dilution used, antibodies against it are referred to as being Gag, Pol-specific. Serial dilutions of samples and previously described macaque serum standards [52] were reacted overnight at 4°C with coated/blocked plates. Plates were developed by treatment with biotinylated polyclonal goat anti-human IgG (SouthernBiotech, Birmingham, AL) or–monkey IgA (Open Biosystems, Huntsville, AL), followed by avidin peroxidase, and tetramethylbenzidine (Sigma). Total IgA and IgG concentrations in secretions were measured by ELISA as previously described [54]. Concentrations of SIV Env- or SIV Gag, Pol-specific IgA and IgG were divided by the concentration of total IgA and IgG, respectively, to obtain the specific activity (ng IgA or IgG antibody per μg total IgA or IgG).

The avidity of anti-Env antibodies in serum was measured using plates coated with gp130 and a NaSCN displacement ELISA modeled after that described by Vermont et al. [55]. The avidity index was calculated by dividing the concentration of anti-gp140 IgG or IgA measured in 1.5M NaSCN-treated wells by that in untreated wells on the same plate.

### Measurement of neutralizing antibodies

Antibody-mediated neutralization of T-cell-line-adapted SIVmac251 (TCLA SIV251) was assessed in a CEMx174 cell killing assay as previously described [16]. Cell-free stocks of TCLA SIV251 prepared in H9 cells were added in triplicate to multiple dilutions of test serum in 100 μl of RPMI 1640–12% fetal bovine serum containing 50 μg gentamicin in 96-well culture plates. After incubation for 1 h at 37°C, CEMx174 cells (5 × 10^4^ cells in 100 μl) were added to each well. Infection led to extensive syncytium formation and virus-induced cell killing in approximately 4 to 6 days in the absence of antibodies. Neutralization was measured by staining viable cells with Finter's neutral red in poly-l-lysine-coated plates. The percent protection was determined by calculating the difference in absorption (*A*540) between test wells (cells plus serum sample plus virus) and virus control wells (cells plus virus), dividing this result by the difference in absorption between cell control wells (cells only) and virus control wells, and multiplying the result by 100. Neutralization was measured at a time when virus-induced cell killing in virus control wells was >70% but <100%. Neutralizing titers are given as the reciprocal dilutions required to protect 50% of cells from virus-induced killing.

### Polychromatic flow cytometry analyses and tetramer staining

Surface staining was carried out by standard procedures for our laboratory as described [56]. Except where noted, all reagents were obtained from BD Biosciences (San Diego, CA) and included monoclonal antibodies to the following molecules: CD3 (clone SP34-2, APC-Cy7 conjugate) CD4 (clone SK3, PerCP-Cy5.5 conjugate), CD8α (clone RPA-T8, Alexa700 conjugate) CD28 (clone CD28.2, PE-Texas Red conjugate, Beckman-Coulter, Fullerton, CA), CCR7 (clone 150503, Pacific Blue conjugate, custom) CXCR3 (clone 1C6, PE-Cy5 conjugate), Ki-67 (Clone B56, PE conjugate), CD127 (clone R34.34, PE conjugate, Beckman-Coulter), perforin (clone Pf-344, FITC conjugate, Mabtech, Mariemont, OH). Intracellular staining for perforin expression was performed using Caltag Fix & Perm (Invitrogen, Camarillo, CA) according to the manufacturer’s suggested protocol. Enumeration of SIV-specific cells using PE- or APC-conjugated pentamers to Mamu-A*01 Gag_181-189_CM9 and Tat_28-35_SL8 (Proimmune, Oxford, UK) was performed as described previously [57]. All samples were analyzed using an LSR II (BD Biosciences), and analyses were performed using FlowJo software (Tree Star Inc., Ashland, OR). Isotype-matched controls and/or fluorescence-minus-one (FMO) controls were included in all assays [58].

### Analysis of SIV-specific cytokine production

Intracellular cytokine staining (ICS) was performed using methods optimized for detection of SIV-specific responses in rhesus macaques [59–61]. Briefly, thawed cryopreserved PBMC were stimulated with SIVmac239 Gag peptides (2 μg/ml) for 12 hours at 37°C in the presence of the co-stimulatory antibodies anti-CD28 and anti-CD49d. GolgiPlug (5 μg/ml), GolgiStop (0.7 μg/ml) and FITC-conjugated antibodies (5x concentration) to the lysosomal degranulation marker, CD107a (clone H4A3), were also added for the duration of stimulation. Media-only and SEB-stimulated cultures served as negative and positive controls, respectively. After culture, cells were surface-stained with fluorochrome-conjugated antibodies to CD4 and CD8 as described above. Cells were subsequently fixed and permeabilized using Caltag Fix and Perm, then incubated for 15 minutes at room temperature in the dark with APC-Cy7-conjugated anti-CD3, PE-conjugated anti-IL-2 (clone MQ1-17H12), PE-Texas Red-conjugated anti-CD69 (clone TP1.55.3, Beckman-Coulter), PE-Cy7-conjugated anti-IFN-γ (clone 4SB3), APC-conjugated anti-TNF-α (clone MAb11). Samples were washed and fixed with 1% freshly prepared paraformaldehyde for at least 1 hour and then analyzed using an LSR II within 24 hours. Lymphocytes were gated based on forward-versus-side scatter characteristics, and the proportions of CD107a- and cytokine-expressing cells were determined by coexpression of CD69 on both CD3^+^CD4^+^ and CD3^+^CD8^+^ lymphocytes using FlowJo v8.8.6 software. All reported values are Gag-specific responses background subtracted from media controls. Multifunctional analyses were performed using Spice v5.32 [62].

### Statistical analysis

All statistical analyses were performed using GraphPad Prism software (GraphPad Software v6.0b, Inc., La Jolla, CA, USA). Spearman’s rank correlation coefficients were used for assessing all correlations. Nonparametric Wilcoxon and Mann–Whitney tests were used for statistical analysis where the sample size was less than or equal to 6. Otherwise, parametric t tests were conducted for quantitative outcomes that are approximately normally distributed; p values less than 0.05 were assumed to be significant in all analyses.

Linear mixed effects models were used to evaluate longitudinal post-challenge viral loads of the three experiment groups compared to the control group. The linear mixed model allows for comparison between different vaccination groups based on the fixed effect of the vaccination duration (5, 20 or 40 weeks) as well as random effects (animal-specific effects), while properly handling serial correlations among repeated viremia measures on the same experimental animal. In this analysis, post-challenge period was classified into three phases over the course of viral infection: peak viremia, viral set-point (weeks 5 to 12) and chronic infection (weeks 13 to 22). Between-group differences in viremia in these three phases were evaluated.

## Supporting Information

S1 Fig Higher antibody response titers and viral load in SIVΔnef-vaccinated Mamu-A*01- animals. (A, B) SIVΔnef-vaccinated Mamu-A*01- animals generated significantly higher levels of serum anti-Env IgG (p = 0.0156) and IgA antibodies (p = 0.0313) than Mamu-A*01+ animals. (C) SIVΔnef-vaccinated Mamu-A*01- animals exhibited significantly higher plasma viral loads than Mamu A*01- animals (p = 0.0005). Two-tailed Wilcoxon tests were used for all statistical analyses.
(EPS)

S2 Fig SIVΔnef antigenic load directly correlates with serum TCLA neutralizing antibody titers. SIVΔnef cumulative plasma viral load, calculated by analyzing the area under the curve of plasma viremia from week four until the end of the vaccination period, directly correlates with the concentration of TCLA neutralizing antibodies in serum (p = 0.0009). SIVΔnef cumulative plasma viral load directly correlates with the concentration of TCLA neutralizing antibodies in serum within group 20 (p = 0.031) and group 40 (p = 0.029) (two-tailed Spearman correlation).
(EPS)

S3 Fig Anti-Env IgA antibody titers do not correlate with post-challenge peak viremia. (A) Anti-Env IgA antibody titers in the serum at day of challenge showed no relationship to peak SIVmac251 viremia post-challenge. (B) Anti-Env IgA specific activity in the vaginal mucosa at day of challenge did not correlate with peak SIVmac251 viremia post-challenge.
(EPS)

S4 Fig No decrease in the CD4 T cell population of the gut mucosa after SIVmac251 challenge. (A) No decrease of the memory CD4 T cell population (CD95+CD4+) as a percentage of total CD3+ T cells in the gut, including the jejunum, the colon and the mesenteric lymph nodes. (B) No difference in the memory CD4 T cell population as a percentage of total CD3+ T cells in the gut of sterilely protected (uninfected) and partially protected (infected) animals at day 14 post-challenge with SIVmac251.
(EPS)

S5 Fig Low proliferation of SL8-specific CD8 T cells at week 20 post-SIVΔnef vaccination. SL8-specific CD8 T cells express significantly lower Ki-67 (p<0.0045) in peripheral blood, secondary lymphoid tissues and the gut mucosa at week 20 than at week 5 (two-tailed unpaired t test).
(EPS)

S1 Table MHC class I genotypes of longitudinal study animals. MHC class I alleles were determined by sequence specific PCR [47].
(DOCX)
