# Supplementary material for: Persistent Low-Level Replication of SIVΔnef Drives Maturation of Antibody and CD8 T Cell Responses to Induce Protective Immunity against Vaginal SIV Infection
Source: PLoS Pathog. 2016 Dec 13;12(12):e1006104. doi: 10.1371/journal.ppat.1006104 (PMC5189958; doi:10.1371/journal.ppat.1006104)
Supplement: S1 Table — MHC class I alleles were determined by sequence specific PCR [47]. (DOCX) [file ppat.1006104.s006.docx]

|  | **Animal** | **Vaccination** | **Group** | **A*01** | **A*02** | **B*08** | **B*17** |  |
| --- | --- | --- | --- | --- | --- | --- | --- | --- |
|  | 102-98 | SIV∆nef | 5 | + | - | - | - |  |
|  | 187-00 | SIV∆nef | 5 | + | - | - | - |  |
|  | 272-00 | SIV∆nef | 5 | - | + | - | + |  |
|  | 127-97 | SIV∆nef | 5 | - | - | - | - |  |
|  | 323-97 | SIV∆nef | 5 | - | - | - | - |  |
|  | 338-96 | SIV∆nef | 5 | - | - | - | - |  |
|  |  |  |  |  |  |  |  |  |
|  | 201-02 | SIV∆nef | 20 | + | - | - | - |  |
|  | 206-00 | SIV∆nef | 20 | + | - | - | - |  |
|  | 408-00 | SIV∆nef | 20 | - | - | - | + |  |
|  | 320-97 | SIV∆nef | 20 | - | + | - | - |  |
|  | 376-02 | SIV∆nef | 20 | - | - | - | - |  |
|  | 410-00 | SIV∆nef | 20 | - | - | - | - |  |
|  |  |  |  |  |  |  |  |  |
|  | 286-07 | SIV∆nef | 40 | + | - | - | - |  |
|  | 292-07 | SIV∆nef | 40 | + | - | - | + |  |
|  | 256-00 | SIV∆nef | 40 | - | + | - | + |  |
|  | 211-02 | SIV∆nef | 40 | - | - | - | - |  |
|  | 225-97 | SIV∆nef | 40 | - | - | - | - |  |
|  | 290-07 | SIV∆nef | 40 | - | - | - | - |  |
|  |  |  |  |  |  |  |  |  |
|  | 401-00 | Control |  | + | - | - | - |  |
|  | 289-07 | Control |  | - | - | - | - |  |
|  | 221-02 | Control |  | - | + | - | - |  |
|  | 100-98 | Control |  | + | - | - | - |  |
|  | 288-03 | Control |  | - | - | - | + |  |
|  | 291-04 | Control |  | - | - | - | - |  |
|  | 321-04 | Control |  | + | - | - | - |  |
|  | 120-05 | Control |  | - | - | - | + |  |
|  | 151-00 | Control |  | - | - | - | - |  |
|  |  |  |  |  |  |  |  |  |
|  |  |  |  |  |  |  |  |  |
